# Knowledge and Prevalence of Risk Factors for Coronary Artery Disease in Patients after Percutaneous Coronary Intervention and Coronary Artery Bypass Grafting

**DOI:** 10.3390/healthcare10061142

**Published:** 2022-06-20

**Authors:** Mikołaj Matysek, Krzysztof Wójcicki, Tomasz Tokarek, Artur Dziewierz, Tomasz Rakowski, Stanisław Bartuś, Dariusz Dudek

**Affiliations:** 1 Department of Cardiology and Cardiovascular Interventions, University Hospital, 31-501 Kraków, Poland; k.j.wojcicki@gmail.com (K.W.); adziewierz@gmail.com (A.D.); mbbartus@cyfronet.pl (S.B.); 2 Center for Invasive Cardiology, Electrotherapy and Angiology, 33-300 Nowy Sacz, Poland; tomek.tokarek@gmail.com; 3 2nd Department of Cardiology, Jagiellonian University Medical College, 31-501 Kraków, Poland; mcrakows@cyf-kr.edu.pl (T.R.); mcdudek@cyfronet.pl (D.D.)

**Keywords:** coronary artery disease, patient knowledge, secondary prevention, lifestyle

## Abstract

Background: Percutaneous coronary intervention (PCI) is associated with a short hospital stay and fast recovery. However, it might be related to insufficient implementation of lifestyle changes after the procedure. Conversely, coronary artery bypass grafting (CABG) is a highly invasive technique that requires a prolonged hospital stay and long rehabilitation with more opportunities for education. This study aimed to evaluate the impact of CABG on adherence to lifestyle modifications and knowledge about coronary artery disease (CAD) in comparison with PCI. We also evaluated the level of education and tried to define groups of patients that might require targeted education. Methods: Data was collected using a self-designed 56-item questionnaire. Questions assessed the knowledge of CAD risk factors and the level of their control. Results: The study group consisted of 155 consecutive patients admitted to the Cardiology Department. Patients with a history of PCI (68%) (at least 8 weeks before) were included in the prior-PCI group, and patients with previous surgical revascularization (also at least 8 weeks before) were assigned to the prior-CABG group (32%). The knowledge score was higher in the prior-CABG group. The median (IQR) results in the prior-PCI vs. prior-CABG group were, respectively: 20 (12–24) vs. 22 (19–25) [points, per 31 max.]; *p* = 0.01. Similar results were obtained in the level of risk control (prior-PCI vs. prior-CABG, respectively: 6 (4–7) vs. 7 (6–8) [points, per 15 max.]; *p* = 0.002). Conclusions: The method of treatment of CAD might impact the implementation of lifestyle modifications after the procedure. More effort is required to improve secondary prevention, especially in PCI patients.

## 1. Introduction

The importance of cardiovascular disorders (CVD), being the leading cause of death in developed countries, is unquestionable [1]. Furthermore, CVD are strongly related to several well-known modifiable risk factors. According to World Health Organization data, up to 80% of premature CVD-related deaths could be avoided with proper prevention introduced in time [2]. Previously reported studies have indicated no differences in the level of knowledge about CVD and risk factor control between patients with a history of one or several percutaneous coronary interventions (PCI) [3,4]. A short hospitalization period or low awareness about the severity of the disease among patients undergoing fast and convenient procedures might explain this finding. On the contrary, coronary artery bypass grafting (CABG) is a more stressful procedure associated with a longer hospital stay. There is a paucity of data comparing adherence to secondary prevention recommendations in patients with a history of several PCI procedures and patients after CABG. Further investigation might provide additional data to understand the factors that shape patients’ attitudes towards lifestyle changes and help create personalized educational programs. Thus, we sought to assess the knowledge, awareness, and prevalence of self-reported risk factors for CAD in the groups of patients after CABG or PCI procedures.

## 2. Materials and Methods

### 2.1. Methodology

**Figure 1:**
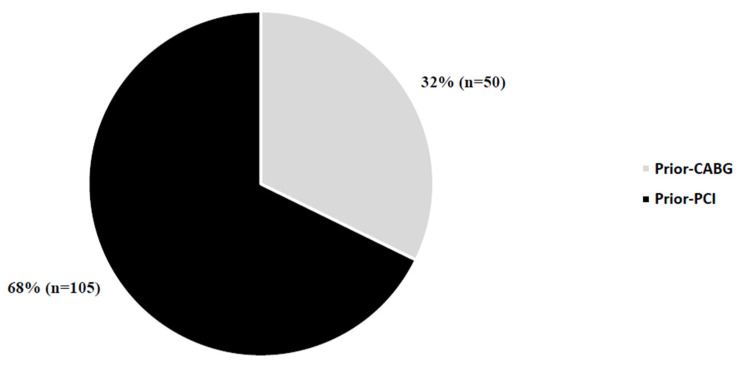
Number of patients in study groups. Abbreviations: CABG, coronary artery bypass grafting; PCI, percutaneous coronary intervention.

A complete description of the methodology was reported previously [3]. Briefly, a self-designed questionnaire comprising 18 questions concerning sociodemographic and clinical profile, 11 assessing knowledge about CAD, and 6 referring to the CAD risk factor control was used. Some questions included a few subsections, so the maximal knowledge score was 31 points. For the assessment of risk control, we analysed information from our survey and clinical data obtained during hospitalization and the maximal score was 15 points. The questionnaire is presented in Supplementary Materials. The questionnaire S1 is presented in Supplementary Materials. The study group included 155 consecutive patients admitted to the 2nd Department of Cardiology and Cardiovascular Interventions at the University Hospital in Kraków (Poland) from July 2016 to July 2019. The inclusion criteria comprised the history of PCI or CABG and the patient’s consent. All patients were enrolled by a trained researcher and signed informed consent and consent for the processing of personal data. All patients provided written informed consent to participate in the study. According to the clinical profile, the patients were divided into two subgroups: the prior-PCI group comprised patients that had undergone PCI at least eight weeks prior to the current hospitalization; the prior-CABG group included patients with a history of CABG at least eight weeks prior to the enrolment. The number of patients in the groups is presented in Figure 1. The study protocol was approved by the local ethics committee. The study was conducted under the ethical principles of clinical research based on the Declaration of Helsinki with its later amendments.

### 2.2. Statistical Analysis

Standard statistical tests performed with Statistica v13 software (StatSoft, Inc., Kraków, Poland) were used for data processing. The Shapiro–Wilk test was used to determine the normality of distribution. The chi-square test was applied for the comparison of qualitative variables, presented as numbers and percentages. Student’s t-test and the Mann–Whitney U test were used for comparison of quantitative variables that were presented as mean and standard deviation (SD) or median and interquartile range (IQR). The correlations between independent variables were assessed with Spearman’s rank correlation, and multiple regression analysis was applied for more complex interaction models. Statistical significance was set at a *p* value < 0.05.

## 3. Results

The results of the comparison of the sociodemographic and clinical profiles are presented in Table 1 and Table 2. The knowledge score was higher in the prior-CABG group (prior-PCI vs. prior-CABG, respectively: 20 (12–24) vs. 22 (19–25) [points, per 31 max.]; *p* = 0.01). Similar results were obtained from the comparison of the level of risk control (prior-PCI vs. prior-CABG, respectively: 6 (4–7) vs. 7 (6–8) [points, per 15 max]; *p* = 0.002). The median age of patients (IQR) was higher in the prior-CABG group: (prior-PCI vs. prior-CABG, respectively: 66 (60–75) vs. 70 (66–79); *p* = 0.03). The median duration of CAD (IQR) was also longer in the prior-CABG group: (prior-PCI vs. prior-CABG, respectively: 8 (3–15) vs. 19 (10–24.5); *p* = 0.001) as well as the proportion patients with a history of two or more previous cardiac hospitalizations was greater in the prior-CABG group (prior-PCI vs. prior-CABG, respectively: 63 (60%) vs. 40 (80%); *p* = 0.02).

The results of the prevalence of particular CAD risk factors are presented in Table 3. Patients after CABG were found to have better control of glucose despite an even higher prevalence of diabetes mellitus (Table 2 and Table 3). In addition, they participated in cardiac rehabilitation more often as compared with the prior-PCI group (Table 3). Stationary rehabilitation was the most popular option. It was attended by 33% of respondents, most often in the prior-CABG group (prior-CABG vs. prior-PCI: 73% vs. 33%; *p* = 0.001). No differences were observed in other forms of rehabilitation. In multiple regression analysis, the impact of cardiac rehabilitation on the level of knowledge score was found only in the prior-CABG group for not attending any form of rehabilitation (R^2^ = 0.2, ꞵ = −9.9; *p* = 0.02). No relationship was found between the level of knowledge and any form of rehabilitation. Avoiding cardiac rehabilitation was associated with worse results in the risk control score in the overall population (R^2^ = 0.20, ꞵ = −2.9; *p* = 0.001) and the prior-PCI group (R^2^ = 0.15, ꞵ = −2.5; *p* = 0.004). Furthermore, participation in ambulatory rehabilitation also appeared to be related to a worse level of risk control in the overall population (R^2^ = 0.19, ꞵ = −1.4; *p* = 0.01) and in the prior-CABG group (R^2^ = 0.3, ꞵ = −3.0; *p* = 0.004). No association was found for any other form of rehabilitation. Prescribed medications were omitted at least once per month by 13% of patients (prior-PCI vs. prior-CABG, respectively: 13% vs. 11%; *p* = 0.7). The comparison of home blood pressure control also revealed no significant differences, 4% of patients never measured blood pressure at home (3% including only hypertensive patients) and 75% made the measurement more than once a week (78% of hypertensive patients). All of the patients in the CABG group controlled blood pressure, even without an original diagnosis of arterial hypertension. The analysis of the frequency of general practitioner (GP) visits revealed no differences between groups. However, patients in the prior-CABG group were found to visit cardiologists more often (visits every six months, prior-CABG vs. prior-PCI: 88% vs. 68%; *p* = 0.01). The risk control score was affected only by the frequency of cardiologist visits and no impact was found for the GP visits; patients visiting cardiologists at least once per 6 months achieved better results: 6 (5–8) vs. 5 (4–6) [points]; *p* = 0.006.

Analysis of the impact of other factors on the level of knowledge and the risk control is presented in Table 4 and Table 5. Significant correlations were found in the overall population for the duration of CAD and the level of risk control (R = 0.17; *p* = 0.002) and the number of hospitalizations and the level of CAD risk control (R = 0.2; *p* = 0.001). Analysis of the relationship between the actual level of patients’ knowledge and self-assessed level of knowledge revealed a correlation in the overall population (R = 0.24; *p* = 0.01) and in the prior-PCI group (R = 0.34; *p* = 0.001), however, a similar correlation was not observed between the self-assessed level of care about one’s health and the level of risk control (R = 0.14; *p* = 0.08).

## 4. Discussion

This study demonstrates that patients after CABG have a higher knowledge score and level of risk control score in comparison with the prior-PCI group. They also more often attended any form of cardiac rehabilitation. However, the age of patients was higher, the duration of the CAD was longer and the number of patients with a history of two or more previous cardiac hospitalizations was also higher in the prior-CABG group. Significant correlations were found in the overall population for the duration of CAD and the level of risk control, and the number of hospitalizations and the level of CAD risk control. These variables and correlations are worth emphasizing and might, from presuppositions, impact higher knowledge and level of risk control scores in the prior-CABG group. Nonetheless, the results of this study suggest that further improvement in education and control of risk factors must be taken into consideration. Treatment of CAD has significantly developed in recent decades, which may result in the reduction of major risk factors [5,6]. The role of the pharmacological treatment of CAD is unquestionable but highly insufficient to obtain maximal control of risk factors [7,8,9,10]. Furthermore, secondary prevention programs have a beneficial impact on risk factor control and both cardiac mortality and the quality of life [11,12]. 

The large cross-sectional study EUROASPIRE IV has shown that most patients with CAD do not fulfil the guideline standards for secondary prevention. A high prevalence of risk factors such as persistent smoking, unhealthy diet, physical inactivity, obesity, and diabetes was observed [7]. Polish multicentre national health survey WOBASZ II has shown that about one-third of the adult population had comorbid hypertension and hypercholesterolemia, and that control of these factors was strongly associated with CAD [13]. Furthermore, less than half of the patients took part in any kind of cardiac prevention and rehabilitation program [7,8,9]. Percutaneous coronary intervention is associated with faster recovery, immediate relief of symptoms, shorter hospital stays, and improved short- medium-term prognosis as compared with CABG [14,15]. The risk of periprocedural death, stroke, and bleeding complications after the PCI procedure is also determined by the access type and the experience of the operator [16]. Stent type might also influence long-term mortality, especially in patients presenting with STEMI [17]. On the contrary, the surgeon’s experience was not associated with long-term mortality following CABG [18]. Another important factor reducing patients’ predicted morbidity and mortality is cardiac rehabilitation [14]. A previous meta-analysis reported reduced cardiovascular mortality and improved quality of life in patients participating in cardiac rehabilitation [19]. Furthermore, it might also be connected with a reduction in the number of uncontrolled risk factors [20]. Different studies have confirmed the importance of cardiac rehabilitation as a part of secondary prevention strategies and demonstrated a reduction in CVD mortality and better quality of life [7,21,22]. The general uptake of cardiac rehabilitation in our study was 56% and it was similar to that reported in other studies [7,9,23,24]. In the prior-CABG group, this percentage was significantly higher (78%) than in the prior-PCI group (46%). Patients after CABG more frequently attend stationary rehabilitation. Cardiac rehabilitation reduces mortality in PCI and CABG patients. However, a greater impact on reducing the number of cardiovascular events was reported in the CABG group [25]. Furthermore, the postprocedural hospital stay is longer after CABG than after PCI [5]. This might be connected to better education provided during post-operative hospitalization and early in-hospital rehabilitation. Further cardiac rehabilitation emphasizes this effect and improves patient awareness in combination with comprehensive education programs [26,27,28]. 

In our institution, patient education includes recommendations written on a discharge card and explanations provided by a medical doctor. Patients are also advised on other reliable sources of information, including online materials. However, lack of time and stress associated with hospitalization might result in poor compliance and a lower level of patient knowledge. 

Higher social status (higher education, income, living in an urban area) was associated with better knowledge results but not with a better risk control score. The SPIRR CAD study examined the standard and psychosocial risk factor profiles concerning socio-economic status (SES) [29]. Only smoking was more common in patients with low SES. The results demonstrated no direct influence of SES on better risk factor control [30]. It might be assumed that education alone does not have the desired impact on the patients’ lifestyle modification. Other programs improving patients’ awareness and motivation should be implemented. In this study, patients after CABG were found to have better control of glucose despite an even higher prevalence of diabetes mellitus. The modifiable risk profiles and risk factor knowledge were improved alternatively in the CHOICE trial [27]. This program comprised clinic visits, telephone support, and obligatory cholesterol reduction and preferential risk factor modification. The CHOICE group had higher risk factor levels such as total cholesterol, systolic blood pressure, smoking status, and physical activity than the control group after one year. Three or more risk factors above the recommended values were reported in 21% of participants. Various educational programs during recent years have proven their efficacy in the improvement of lifestyle changes [26,28,31,32]. The correlation between the level of knowledge and the level of risk factor control has not been found in the overall population. Patients achieving better results in knowledge tests had no improvement in CAD risk factor control. These findings are in line with data suggesting no impact of education alone on the mortality rate [11]. Thus, personalized educational programs for CAD patients might be crucial for further improvement in the long-term outcomes.

## 5. Conclusions

Patients after CABG have a higher knowledge score and level of risk factor control score in comparison to the patients after PCI. Longer procedure-related hospitalization, a greater number of hospitalizations, a longer duration of CAD, and the scope of the CABG procedure might be associated with these findings. Education and cardiac rehabilitation programs should be widely implemented in patients with CAD.

### Study Limitations

Several limitations should be acknowledged. We presented data based on a single-centre experience with a relatively low number of patients. We have only included patients admitted to the 2nd Department of Cardiology and Cardiovascular Interventions at the University Hospital in Kraków (Poland) from July 2016 to July 2019. Therefore, multicentre studies are essential to creating recommendations and educational programs that might be introduced into common clinical practice. Furthermore, there are several significant differences in the clinical profiles between the main groups of the study. Although, we were focused not only on the impact of the CABG procedure itself but also on the profile of patients in the study groups to identify factors with the potential to impact knowledge and risk control. All included patients were stable and admitted with chronic coronary artery syndrome, but also with various cardiological comorbidities. Thus, patients with an initially more severe condition at admission might have had poorer adherence to health recommendations. Furthermore, the health and mental condition might influence the data provided in the questionnaire. Another limitation is the absence of validation. We decided to use a self-constructed questionnaire. However, there is a lack of validated tools that might be suitable for this study. Our study included a relatively low number of patients. Thus, we could not use propensity score matching in statistical analysis. Therefore, an imbalance in baseline characteristics might be observed between the groups.

## Figures and Tables

**Table 1:** Comparison of sociodemographic profile.

Variable		All Patients (*n* = 155)	Prior-PCI (*n* = 105)	Prior-CABG (*n* = 50)	*p* Values
Gender (% of male)		110 (71%)	71 (68%)	39 (78%)	0.2
Age, years [median (IQR)]		68 (62–77)	66 (60–75)	70 (66–79)	0.03
Education	primary, secondary or vocational	124 (80%)	86 (82%)	38 (76%)	0.4
	higher	31 (20%)	19 (18%)	12 (24%)	
Current marital status	married	112 (72%)	76 (72%)	36 (72%)	0.99
	not married	43 (28%)	29 (28%)	14 (28%)	
Place of residence	rural areas	37 (24%)	27 (26%)	10 (20%)	0.4
	city	118 (76%)	78 (74%)	40 (80%)	
Net monthly household income	below EUR 875 *	127 (82%)	89 (85%)	38 (76%)	0.2
	above EUR 875 *	28 (18%)	16 (15%)	12 (24%)	

* The equivalent of PLN 4000 at the current exchange rate.

**Table 2:** Comparison of clinical profile.

Variable	All Patients (*n* = 155)	Prior-PCI (*n* = 105)	Prior-CABG (*n* = 50)	*p* Values
Duration of CAD, years [median (IQR)]	11 (4–20)	8 (3–15)	19 (10–24.5)	0.001
History of two or more previous cardiac hospitalizations	103 (66%)	63 (60%)	40 (80%)	0.02
Previous MI	87 (56%)	64 (61%)	23 (46%)	0.08
Diabetes mellitus	67 (43%)	41 (39%)	26 (52%)	0.1
Hypercholesterolemia	134 (86%)	90 (86%)	44 (88%)	0.8
Arterial hypertension	149 (96%)	101 (96%)	48 (96%)	0.9
Family history of CAD	49 (32%)	36 (34%)	13 (26%)	0.4
Early diagnosis of CAD (<55 years old in men, <65 years in women)	92 (59%)	59 (56%)	33 (66%)	0.2

Abbreviations: CABG, coronary artery bypass grafting; CAD, coronary artery disease; IQR, interquartile range; MI, myocardial infarction; PCI, percutaneous coronary intervention.

**Table 3:** Comparison of particular CAD risk factors in study groups.

Variable	All Patients (*n* = 155)	Prior-PCI (*n* = 105)	Prior-CABG (*n* = 50)	*p* Value
Little physical activity (regular activity < 150 min a week)	113 (73%)	78 (74%)	35 (70%)	0.6
No cardiac rehabilitation	68 (44%)	57 (54%)	11 (22%)	0.001
LDL-C level > 1.8 mmol/L	75 (48%)	50 (48%)	25 (50%)	0.9
Fasting glucose level > 5.5 mmol/L	59 (38%)	49 (47%)	10 (20%)	0.0497
Systolic blood pressure > 140 mmHg	61 (49%)	46 (44%)	15 (30%)	0.1
Obesity (BMI ≥ 30 kg/m^2^)	57 (37%)	41 (39%)	16 (32%)	0.4
Current smoking	33 (21%)	26 (25%)	7 (14%)	0.1
Frequency of GP visits (at least once a month, %)	77 (50%)	50 (48%)	27 (53%)	*p* = 0.2
Frequency of cardiologist visits (at least one in 6 mths)	115 (74%)	71 (68%)	44 (88%)	*p* = 0.001
Measures BP at least once a week:	123 (79%)	81 (77%)	42 (83%)	*p* = 0.07

Abbreviations: BMI, body mass index; CABG, coronary artery bypass grafting; LDL-C, low-density lipoprotein cholesterol; PCI, percutaneous coronary intervention; GP, general practitioner; BP, blood pressure.

**Table 4:** Impact of factors other than a history of PCI or CABG on the level of knowledge and the level of coronary artery disease risk control—independent analysis.

Variable		Median Level of Knowledge (IQR) (Points, Max. 31)	*p* Value	Median Level of Risk Control (IQR) (Points, Max. 15)	*p* Value
All patients		21 (15–25)		6 (4–8)	
Age	below 65 years	19 (14–23)	0.07	5 (4–7)	0.04
	above 65 years	22 (16–25)		6 (5–8)	
Gender	male	21 (17–24)	0.6	6 (4–8)	0.06
	female	21 (12–25)		6 (4–7)	
Education	primary, secondary or vocational	20 (13–25)	0.05	6 (4–8)	0.7
	higher	23 (20–24)		6 (5–7)	
Marital status	married	21 (16–24.5)	0.5	6 (5–8)	0.2
	not married	21 (14–25)		6 (4–7)	
Place of residence	village	20 (13–25)	0.3	6 (4–7.5)	0.4
	city	22 (17–25)		6 (4–8)	
Net household income	below EUR 875 *	20 (12–24)	0.03	6 (4–7)	0.2
	above EUR 875 *	23 (21–25)		6 (5–8)	
Previous MI	no history of MI	22 (14–25)	0.5	6 (4–7)	0.2
	history of MI	21 (15–24)		6 (5–8)	

Abbreviations: CABG, coronary artery bypass grafting; IQR, interquartile range; MI, myocardial infarction; PCI, percutaneous coronary intervention.* The equivalent of PLN 4000 at the current exchange rate.

**Table 5:** Impact of factors other than a history of PCI or CABG on the level of knowledge and the level of coronary artery disease risk control—multiple regression model.

Variable	Level of Knowledge R^2^ = 0.08		Level of Risk Control R^2^ = 0.08	
	β	*p* Value	β	*p* Value
Constant	4.7	0.4	3.2	0.1
Age (per 1-year increase)	0.1	0.1	0.04	0.01
Gender	0.4	0.8	0.6	0.1
Education	−0.01	1.0	−0.1	0.9
Marital status	−0.8	0.6	−0.1	0.8
Place of residence	1.5	0.3	−0.5	0.2
Net household income	3.8	0.01	0.8	0.1
Previous MI	−0.3	0.8	0.1	0.7

Abbreviations: CABG, coronary artery bypass grafting; MI, myocardial infarction; PCI, percutaneous coronary intervention.

## Data Availability

The data that support the findings of this study are available from the corresponding author upon reasonable request.

## Supplementary Materials

The following supporting information can be downloaded at: https://www.mdpi.com/article/10.3390/healthcare10061142/s1, The questionnaire S1: Assessment of the knowledge and control of risk factors for the development of atherosclerosis among patients after first-time and subsequent revascularization of coronary vessels.
