# Supplementary material for: Knowledge and Prevalence of Risk Factors for Coronary Artery Disease in Patients after Percutaneous Coronary Intervention and Coronary Artery Bypass Grafting
Source: Healthcare (Basel). 2022 Jun 20;10(6):1142. doi: 10.3390/healthcare10061142 (PMC9223287; doi:10.3390/healthcare10061142)
Supplement: Supplementary file 1 [file healthcare-10-01142-s001.zip › healthcare-1731137-supplementary.pdf]

Ocena stanu wiedzy oraz kontroli czynników ryzyka rozwoju miażdżycy wśród pacjentów po pierwszorazowej oraz kolejnej rewaskularyzacji naczyń wieńcowych

PODSTAWOWE DANE PACJENTA

1. **Płeć**
   Zaznacz tylko jedną odpowiedź.
   - kobieta
   - mężczyzna

2. **Data urodzenia**

   Przykład: 15 grudnia 2012

3. **Wzrost [cm]**

4. **Waga [kg]**

5. **Obwód pasa [cm]**

6. **Obwód bioder [cm]**

7. **Miejsce zamieszkania**
   Zaznacz tylko jedną odpowiedź.
   - wieś
   - miasto do 50 tys. mieszkańców
   - miasto 50 - 100 tys. mieszkańców
   - miasto powyżej 100 tys. mieszkańców

8. **Wykształcenie**
Zaznacz tylko jedną odpowiedź.

- podstawowe
- zasadnicze zawodowe
- średnie
- wyższe- licencjat, inżynier
- wyższe- magister
- podyplomowe

9. **Aktywność zawodowa**
Zaznacz tylko jedną odpowiedź.

- aktywny zawodowo - praca fizyczna
- aktywny zawodowo - praca umysłowa
- uczeń
- emeryt - dawniej praca fizyczna
- emeryt - dawniej praca umysłowa
- rencista
- bezrobotny

10. **Stan cywilny**
Zaznacz tylko jedną odpowiedź.

- zamężna / żonaty
- panna / kawaler
- rozwódka/ rozwodnik
- wdowa / wdowiec

11. **Ile osób wchodzi w skład Państwa gospodarstwa domowego?**

....

12. **W którym z poniższych przedziałów mieszczą się miesięczne dochody brutto Państwa gospodarstwa domowego?**
Zaznacz tylko jedną odpowiedź.

- poniżej 2500 zł
- 2500 - 4000 zł
- 4000 - 6000 zł
- powyżej 6000 zł

13. **Uzyskiwane dochody określiłaby Pani/Pan jako:**
Zaznacz tylko jedną odpowiedź.

- Niskie, niewystarajace
- Odpowiednie, wystarczajace
- Wysokie

14. **Jak często chodzi Pani/Pan na wizyty kontrolne do wymienionych lekarzy?**
Zaznacz tylko jedną odpowiedź w rzędzie.

	częściej niż raz w miesiącu	raz w miesiącu	raz na 3 miesiące	raz na 6 miesięcy	raz na rok	rzadziej niż raz w roku
Lekarz rodzinny						
Lekarz kardiolog						

15. **Czy przed obecnym pobytem w szpitalu byli Państwo hospitalizowani z powodu choroby serca/ na oddziale kardiologicznym? Jeśli tak, to ile razy?**

....

16. **Kiedy rozpoznano u Pani/Pana chorobę niedokrwienną serca? (w miesiącach lub latach)**

....

17. **Czy podczas pobytu na oddziale kardiologicznym zapewniono Pani/Panu edukację z zakresu czynników ryzyka choroby niedokrwiennej serca?**
Zaznacz tylko jedną odpowiedź.

- tak, przy każdym pobycie w szpitalu
- tak, ale nie przy każdym pobycie w szpitalu
- nie
- nie pamiętam

18. **Używki (podać ilość i czas trwania nałogu)**
Zaznacz tylko jedną odpowiedź w rzędzie.

	papierosy	alkohol	kawa
teraz			
w przeszłości			

WIEDZA PACJENTA

19. **Jak ocenia Pani/Pan własną wiedzę na temat czynników ryzyka choroby wieńcowej?**
Zaznacz tylko jedną odpowiedź.

	1	2	3	4	5	
nie mam żadnej wiedzy						mam pełną wiedzę

20. **Z jakich źródeł czerpie Pani/Pan wiedzę na temat tego, w jaki sposób żyć po zawale serca?**

Zaznacz wszystkie właściwe odpowiedzi.

- [ ] uczestnictwo w Klubie Pacjenta/ Szkole Serca
- [ ] od lekarza pierwszego kontaktu
- [ ] od lekarza kardiologa
- [ ] od rodziny, znajomych
- [ ] z TV
- [ ] z książek, prasy, broszur informacyjnych
- [ ] z internetu
- [ ] nie szukam informacji na ten temat
- [ ] Inne: ....

21. **Proszę określić w jakim stopniu wymienione czynniki wpływają na zachorowanie na chorobę wieńcową**

Zaznacz tylko jedną odpowiedź w rzędzie.

	Nie wpływa	Wpływa	Nie wiem
Nadciśnienie tętnicze	○	○	○
Cukrzyca	○	○	○
Wysoki poziom cholesterolu	○	○	○
Palenie papierosów	○	○	○
Otyłość	○	○	○
Mała aktywność fizyczna, siedzący tryb życia	○	○	○
Dieta bogata w tłuszcze zwierzece	○	○	○
Wiek	○	○	○
Płeć	○	○	○
Predyspozycje rodzinne	○	○	○
Nieskie dochody, niskie wykształcenie	○	○	○
Wysoki poziom stresu w życiu	○	○	○
Depresja, ograniczenie kontaktów społecznych	○	○	○

22. **Czy wyeliminowanie przynajmniej jednego czynnika ryzyka miażdżycy może mieć pozytywny wpływ na rozwój choroby wieńcowej?**

Zaznacz tylko jedną odpowiedź.

- ○ Tak, choć celem powinno być wyeliminowanie wszystkich (usunięcie jednego ułatwi eliminację kolejnych)
- ○ Tak, wystarczy wyeliminować jeden, najgroźniejszy czynnik
- ○ Nie, eliminacja pojedynczego czynnika ryzyka nie przekłada się na lepsze rokowanie
- ○ Nie wiem

23. **Jakie działania należy podejmować, aby kontrolować podane czynniki ryzyka chorób serca i naczyń?**
Zaznacz tylko jedną odpowiedź w rzędzie.

	Stosowanie leków	Zdrowa dieta	Regularne ćwiczenia fizyczne	Zaprzestanie palenia	Żadne, nic nie można zrobić	Nie wiem
Nadciśnienie tętnicze	◯	◯	◯	◯	◯	◯
Cukrzyca	◯	◯	◯	◯	◯	◯
Hipercholesterolemia	◯	◯	◯	◯	◯	◯
Utrzymanie lub osiągnięcie prawidłowej masy ciała	◯	◯	◯	◯	◯	◯

CIŚNIENIE TĘTNICZE KRWI

24. **Proszę określić jakich wartości ciśnienie tętnicze nie powinno przekraczać**
Zaznacz tylko jedną odpowiedź.

- ◯ 120/80
- ◯ 140/90
- ◯ Podczas aktywności / w stresie dopuszczalne jest ciśnienie powyżej 140/90
- ◯ Nie ma określonej granicy, prawidłowe ciśnienie to takie, przy którym pacjent czuje się dobrze
- ◯ Nie wiem

POZIOM GLUKOZY WE KRWI

25. **Proszę podać prawidłowy poziom glukozy we krwi na czczo:**
Zaznacz tylko jedną odpowiedź.

- ◯ 50 - 70 mg% (2,8 – 2,9 mmol/l)
- ◯ 70 - 99 mg% (3,9 - 5,5 mmol/l)
- ◯ 100 - 125 mg% (5,6 - 6,9 mmol/l)
- ◯ 126 - 150 mg% (7,0 – 8,3 mmol/l)
- ◯ nie wiem

POZIOM CHOLESTEROLU WE KRWI

26. **Na który parametr w lipidogramie należy zwrócić największą uwagę, ponieważ ma on największe znaczenie w powstawaniu miażdżycy?**
Zaznacz tylko jedną odpowiedź.

- ◯ Cholesterol LDL
- ◯ Cholesterol HDL
- ◯ Trójglicerdy
- ◯ nie wiem

MASA CIAŁA

27. **Które z określeń najlepiej pasuje do Pani/Pana obecnej masy ciała?**
Zaznacz tylko jedną odpowiedź.

- Niedowaga
- Waga prawidłowa
- Nadwaga
- Otyłość
- Skrajna otyłość

28. **Jaka jest Pani/Pana zdaniem główna przyczyna Pani/Pana zbyt wysokiej wagi?**
Zaznacz wszystkie właściwe odpowiedzi.

- [ ] nie cierpię na nadwagę/otyłość
- [ ] wrodzona skłonność
- [ ] stres
- [ ] zbyt mała aktywność fizyczna
- [ ] nieodpowiednia dieta
- [ ] skutek uboczny stosowanych leków
- [ ] nie wiem

STYL ŻYCIA

29. **Jaki Pani/Pana zdaniem wpływ stylu życia na ryzyko wystąpienia choroby niedokrwiennej serca ?**
Zaznacz tylko jedną odpowiedź.

- żaden
- mały
- duży
- nie wiem

30. **Po jakim czasie u chorych po zawale serca dąży się do powrotu do normalnego trybu życia?**
Zaznacz tylko jedną odpowiedź.

- Od razu po wyjściu ze szpitala - jeśli zabieg na naczyniach wieńcowych się udał to jestem zdrowy
- po 6-8 tygodniach
- po 6 miesiącach
- Powrót do normalnego trybu życia nie jest już nigdy możliwy
- nier wiem

UŻYWKI

31. **Czy Pani/Pana zdaniem zaprzestanie palenia ma wpływ na ograniczenie negatywnych skutków choroby wieńcowej, która zdążyła się już ujawnić?**
Zaznacz tylko jedną odpowiedź.

- Tak
- Nie
- Nie wiem

32. **Czy u chorych na chorobę niedokrwienną serca dopuszczalne jest spożycie napojów alkoholowych?**
*10g alkoholu = 275ml piwa 5% = 100ml wina 13% = 30ml wódki 40%
Zaznacz wszystkie właściwe odpowiedzi.

- [ ] Zdecydowanie nie, w żadnej ilości
- [ ] Mała ilość alkoholu (20g u mężczyzn, 10g u kobiet dziennie) jest dozwolona
- [ ] Alkohol - nawet w dużych ilościach - nie ma wpływu na serce
- [ ] Nie wiem

DIETA

33. **Proszę wskazać jakie produkty należy spożywać a jakich należy unikać po zawale serca.**
Zaznacz tylko jedną odpowiedź w rzędzie.

	należy spożywać	należy unikać
sól kuchenna powyżej 5g (łyżeczka)		
produkty zawierające duże ilości błonnika pokarmowego (np. pełnoziarniste pieczywo)		
kwasy tłuszczowe pochodzenia zwierzęcego		
kwasy tłuszczowe pochodzenia roślinnego (np. olej)		
nienasycone kwasy tłuszczowe grupy trans (margaryna, produkty cukiernicze)		
owoce w ilości powyżej 200g dziennie		
ryby co najmniej 2 razy w tygodniu		
tłuste ryby 1 raz w tygodniu		
cukry proste (np. słodycze, miód)		
warzywa w ilości powyżej 200g dziennie		

AKTYWNOŚĆ FIZYCZNA

34. **Co oznacza zalecana pacjentom z chorobą niedokrwienną serca umiarkowana aktywność fizyczna?**
Zaznacz tylko jedną odpowiedź.

- ćwiczenia o małej intensywności (30-45min dziennie), praktykowane regularnie (3-5 razy w tygodniu)
- ćwiczenia o dużej intensywności i długim czasie trwania, bo tylko takie mają wpływ na zdrowie
- żadne ćwiczenia nie są rekomendowane, bo stanowią niebezpieczeństwo dla chorego z chorobą wieńcową
- nie wiem

KONTROLA CZYNNIKÓW RYZYKA

wypełnia pacjent

35. **Generalnie można powiedzieć, że stan Pani/Pana zdrowia jest:**
Zaznacz tylko jedną odpowiedź.

	1	2	3	4	5	
bardzo zły						bardzo dobry

36. **W jakim stopniu choroba serca utrudnia Pani/Panu codzienne funkcjonowanie?**
Zaznacz tylko jedną odpowiedź.

	1	2	3	4	5	
Wcale nie utrudnia						Uniemożliwia mi funkcjonowanie

37. **Jak ocenia Pani/Pan efektywność edukacji na temat życia z chorobą serca prowadzonej przez pracowników służby zdrowia?**
Zaznacz tylko jedną odpowiedź.

	1	2	3	4	5	
bardzo źle, edukacja nieobecna						bardzo dobrze, wyjaśniono mi jak powinno się postępować po zawale

38. **W jakich formach rehabilitacji kardiologicznej Państwo uczestniczyli?**
Zaznacz wszystkie właściwe odpowiedzi.

- [ ] rehabilitacja w trybie stacjonarnym (oddział rehabilitacyjny, uzdrowisko)
- [ ] rehabilitacja w trybie ambulatoryjnym (poradnia rehabilitacji kardiologicznej, oddział dzienny rehabilitacji kardiologicznej)
- [ ] rehabilitacja w domu
- [ ] uczestnictwo w Klubie Pacjenta/ Szkole Serca
- [ ] nie brałem udziału w żadnej formie rehabilitacji

39. **Czy zdarza się Pani/Panu zapomnieć o przyjęciu przepisanego na stałe leku?**
Zaznacz tylko jedną odpowiedź.

- Nie, nigdy mi się nie zdarza
- Rzadziej niż raz w miesicu
- Kilka razy w miesiącu lub częściej
- Zażywam przepisane leki tylko gdy mam dolegliwości

40. **Jakie działania Pani/Pan podejmuje, aby kontrolować podane czynniki ryzyka chorób serca i naczyń?**
Zaznacz tylko jedną odpowiedź w rzędzie.

	Stosowanie leków	Zdrowa dieta	Regularne ćwiczenia fizyczne	Zaprzestanie palenia	Nie podejmuję żadnych działań
Nadciśnienie tętnicze					
Cukrzyca					
Hipercholesterolemia					
Utrzymanie lub osiągnicie prawidłowej masy ciała					

41. **Proszę wybrać, którego zalecenia dotyczącego zapobiegania chorobie wieńcowej jest Pani/Panu najtrudniej przestrzegać?**
Zaznacz tylko jedną odpowiedź.

- Kontrola cukrzycy
- Kontrola ciśnienia tętniczego
- Utrzymanie diety niskokalorycznej i ubogotłuszczowej
- Kontrola masy ciała
- Całkowite zaprzestanie palenia
- Aktywność fizyczna 30-45 minut dziennie, 3-5 razy w tygodniu
- Przyjmowanie wszystkich zaleconych leków (podaj z jakiego powodu):
- Inne:

42. **Proszę zaznaczyć, których zaleceń dotyczących zapobiegania chorobie wieńcowej nie udaje się Pani/Panu przestrzegać?**
Zaznacz wszystkie właściwe odpowiedzi.

- [ ] Kontrola cukrzycy
- [ ] Kontrola ciśnienia tętniczego
- [ ] Utrzymanie diety niskokalorycznej i ubogotłuszczowej
- [ ] Kontrola masy ciała
- [ ] Całkowite zaprzestanie palenia
- [ ] Aktywność fizyczna 30-45 minut dziennie, 3-5 razy w tygodniu
- [ ] Przyjmowanie wszystkich zaleconych leków (podaj z jakiego powodu):
- [ ] Przestrzegam wszystkich zaleceń
- [ ] Inne:

CIŚNIENIE TĘTNICZE

43. **Jak często dokonuje Pani/Pan pomiarów ciśnienia tętniczego?**
Zaznacz tylko jedną odpowiedź.

- Co najmniej 1 raz dziennie
- Raz w tygodniu
- Tylko przed wizyta kontrolną u lekarza
- Tylko w razie złego samopoczucia
- Nie mierze ciśnienia

44. **Jakie wartości ciśnienia skurczowego otrzymuje Pani/Pan zazwyczaj w trakcie domowych pomiarów w czasie spoczynku?**
Ciśnienie skurczowe- np. 120 w przypadku ciśnienia 120/80 ("większa wartość")
Zaznacz tylko jedną odpowiedź.

- Mniejsze niż 140
- 140-159
- 160-179
- pozwyżej 180
- Nie mierzę ciśnienia w domu

DIETA

45. **Ile posiłków dziennie Pani/Pan spożywa?**
Zaznacz tylko jedną odpowiedź.

- mniej niż 3 posiłki dziennie
- 3 do 5 posiłków dziennie
- więcej niż 5 posiłków dziennie

MASA CIAŁA

46. **Jeśli udało się Pani/Panu zredukować masę ciała, to którą metodę określiliby Państwo jako najbardziej pomocną?**
Zaznacz tylko jedną odpowiedź.

- nigdy nie podjąłem (-am) próby redukcji masy ciała (w tym: bo nie mam nadwagi)
- wszystkie podjęte próby redukcji masy ciała były nieskuteczne
- zmiana nieprawidłowych nawyków żywieniowych
- zwiększenie aktywności fizycznej
- leczenie farmakologiczne
- leczenie dietetyczne
- psychoterapia
- leczenie chirurgiczne (operacja bariatryczna)
- Inne: ....

47. **Proszę określić, ile razy podejmowali Państwo nieskuteczną próbę redukcji masy ciała.**

STYL ŻYCIA

48. **Który z czynników miał największy wpływ na to jak wygląda Pani/Pana obecny styl życia?**
Zaznacz tylko jedną odpowiedź.

- Wychowanie i wykształcenie
- Przeżyte choroby i inne trudne wydarzenia życiowe
- Status majątkowy
- Czynniki kulturowe, społeczeństwo w którym żyje
- Wykonywana praca
- Żadne z powyższych

UŻYWKI

49. **Jeśli udało się Pani/Panu rzucić palenie, to którą metodę określiliby Państwo jako najbardziej pomocną?**
Zaznacz tylko jedną odpowiedź.

- Nigdy nie podjąłem (-am) próby rzucenia palenia [w tym: bo nigdy nie paliłem (-am)]
- Wszystkie podjęte próby rzucenia palenia były nieskuteczne
- Silna wola, mocne postanowienie
- Metody farmakologiczne (nikotynowa terapia zastępcza, inne)
- Indywidualna lub grupowa terapia psychologiczna
- inne:

50. **Proszę określić, ile razy podejmowali Państwo nieskuteczną próbę rzucenia palenia?**

....

DIETA

51. **Proszę wybrać ile dziennie porcji następujących produktów Pani/Pan zazwyczaj spożywa**
Zaznacz tylko jedną odpowiedź w rzędzie.

	maksymalnie 1 porcja dziennie	2-3	4-5	6 i więcej porcji dziennie
Produkty zbożowe (np. 1 kromka chleba, 1/2 szklanki płatków)				
Niskotłuszczowe produkty mleczne (np. 1 szklanka mleka/jogurtu, 45g sera białego)				
Mięso, ryby, drób (np. 100g wędliny, kotlet schabowy)				
Owoce (np. 1 jabłko/banan, szklanka soku owocowego)				
Warzywa (np. 1 pomidor, szklanka soku warzywnego)				
Orzechy, rośliny strączkowe (np. garść orzechów, 1/2 szklanki gotowanego grochu)				
Tłuszcze i oleje (np. 1 łyżeczka oleju/margaryny)				
Słodycze (np. 1 łyżeczka cukru, kostka czekolady)				

AKTYWNOŚĆ FIZYCZNA

52. **Ile razy w tygodniu wykonuje Pani/Pan ćwiczenia fizyczne trwające około 30-45 minut?**
Zaznacz tylko jedną odpowiedź.

0	1	2	3	4	5	6	7

CZYNNIKI PSYCHOSPOŁECZNE

53. **Jak opisałby Pan/Pani poziom stresu w swoim życiu?**
Zaznacz tylko jedną odpowiedź.

	1	2	3	4	5	
bardzo niski						wysoki, utrudniajcy codzienne funkcjonowanie

54. **Jak ocenia Pani/Pan poziom własnej troski o zdrowie?**
Zaznacz tylko jedną odpowiedź.

	1	2	3	4	5	
Wcale się tym nie przejmuje						Wypełniam wszystkie zalecenia

55. **Kto pomaga Pani/Panu w przestrzeganiu zaleceń lekarza (podaje leki, dba o odpowiednią dietę)?**

Zaznacz tylko jedną odpowiedź.

- sam się o to troszczę
- rodzina (współmałżonek, dzieci)
- opiekun medyczny lub pielęgniarka środowiskowa

56. **Jaką formę edukacji na temat czynników ryzyka choroby wieńcowej chcieliby Państwo otrzymywać? (np. spotkania z lekarzami, broszury informacyjne i inne)**

....

Technologia Google Forms
